# Oxygen/ozone as a medical gas mixture. A critical evaluation of the various methods clarifies positive and negative aspects

**DOI:** 10.1186/2045-9912-1-6

**Published:** 2011-04-28

**Authors:** Velio Bocci, Iacopo Zanardi, Valter Travagli

**Affiliations:** 1 Dipartimento di Fisiologia, Università degli Studi di Siena, Viale Aldo Moro, 2 - 53100 Siena, Italy; 2 Dipartimento Farmaco Chimico Tecnologico, Università degli Studi di Siena, Viale Aldo Moro, 2 - 53100 Siena, Italy

## Abstract

Besides oxygen, several other gases such as NO, CO, H_2_, H_2_S, Xe and O_3 _have come to age over the past few years. With regards to O_3_, its mechanisms of action in medicine have been clarified during the last two decades so that now a comprehensive framework for understanding and recommending ozone therapy in various pathologies is available. O_3 _used within the determined therapeutic window is absolutely safe and more effective than golden standard medications in numerous pathologies, like vascular diseases. However, ozone therapy is mostly in practitioners' hands and some recent developments for increasing cost effectiveness and speed of treatment are neither standardized, nor evaluated toxicologically. Hence, the aim of this article is to emphasize the need to objectively assess the pros and cons of oxygen/ozone as a medical gas mixture in the hope that ozone therapy will be accepted by orthodox medicine in the near future.

## Introduction

Two decades ago it would have appeared paradoxical to suggest the use of some gases for therapeutic purposes, but today there is an ample literature indicating the importance of NO [1-3], CO [1,3-6], CO_2_[7], H_2_S [1,3,6,8], H_2_[1,9], Xe [1,10], and O_3_[11-13]. Oxygen, although it is essential for aerobic organisms for respiration as well as energy production, has been therapeutically used for a long time [14]. It also can be either toxic or lethal for humans if it is continuously inhaled pure for about 60 hours [15]. In the 16th century Paracelsus had already expressed the concept that "the dose makes the poison". Indeed, minimal concentration of NO, CO and H_2_S are of crucial importance in physiological conditions, but they become detrimental at high doses. Although the transient presence of an ozone-like compound has been postulated in atherosclerotic plaques [16,17], it has no role in normal conditions. Ozone is one of the most controversial gases because it is useful in the stratosphere for blocking UV radiations but toxic in the troposphere during chronic air inhalation. This is because in humans the great expanse of the alveolar surface (about 70 m^2^) is protected only by a scanty volume (about 25 mL) of lining fluid which, by having a small antioxidant content, cannot quench the strong oxidant activity of ozone. The opposite is true for blood because both the plasma and blood cells have a remarkable quantity of mutually cooperating antioxidants. Against the old concept that ozone is always toxic and it should not be used in medicine, a comparative analysis between the lungs vs. blood has fully clarified the possibility of using ozone as a therapeutic agent provided that dosages are not overwhelming the blood antioxidant capacity [18].

The aim of this article is to clarify the state-of-the-art therapeutic use of the oxygen/ozone mixture. The versatility of ozone application is impressive because if properly used it can be proficient in vascular diseases (chronic limb ischemia, diabetic foot, heart infarction and stroke) and surprisingly in orthopedics and odontoiatry [19]. In infection diseases (bacterial, viral, fungal), it can only support antibiotics and/or chemotherapy because ozone cannot ever destroy bacteria or viruses in blood and cells where disappointingly these pathogens are well protected by the endogenous antioxidant system [20]. On the other hand, ozone either as a gas or dissolved in pure water can be an excellent disinfectant for cutaneous and mucosal infections. Nonetheless, it has become clear that well-developed countries neglect or ostracize the use of ozone in medicine because of the ample availability of drugs and the power of pharmaceutical firms. On the other hand, in less-developed countries in places such as South America and Eastern Europe (mainly Russia and Ukraine), expensive drugs are less available and there is a growing tendency to use ozone as a sort of panacea. Owing to the vast number of patients, a worrying trend is the invention of cheap and rapid ozone treatments which are based on approaches which may be either unsafe or ineffective. Apart from the use of topical ozone derivatives in dermatology [21,22], such a concern may get even worse because some practitioners and naturopaths, lacking on appropriate chemical and biological skills, do not understand the toxic risk of unstable solutions, possibly containing excessive amounts of ozone. There are many commercially available medical ozone generators able to monitor the production of ozone as a gas, thus determining the ozone concentration. Any interested reader must be careful on the quality of both equipment materials and measurement devices, mainly in terms of ozone-resistance and accuracy, respectively.

### A) Mechanism of action of ozone in blood

These aspects have been extensively discussed elsewhere [11,23] and they need to be summarized as follows. Owing to the potent antioxidant capacity of blood due to its hydrophilic, lipophilic antioxidants and cellular enzymes, some of the ozone dose dissolved in the water of plasma is instantly quenched by free antioxidants (mainly uric acid, ascorbic acid, reduced glutathione - GSH, cysteine and albumin), while the remaining ozone reacts with polyunsaturated fatty acid (PUFA) mostly present in the three hydrophobic tasks of albumin [24].

Thus, the potential energy of ozone is finally transferred into two fundamental messengers such as H_2_O_2 _as a reactive oxygen species (ROS) and aldehydic molecules of which 4-hydroxynonenal (4-HNE) and trans-4-hydroxyhexenal (4-HHE) are the relevant lipid oxidation products (LOP):

Due to the high ozone reactivity, these biochemical reactions occur in a few seconds and in fact, within the canonical five minutes of mixing an average 200 mL of human blood ex vivo in a sterile glass bottle with the 200 mL corresponding volume of the gas mixture (O_2_+O_3_), ozone is totally exhausted while about 95% oxygen, dissolved in the plasma water, fully saturates hemoglobin.

Blood oxygenation goes up to about 400 mm Hg in the bottle is useful, but it has only a little practical relevance because oxygenated-ozonated blood is normally reinfused via venous route into the donor during the next 20 minutes and is abundantly diluted with venous blood. Therefore ozone represents the medical drug while pure oxygen is only necessary for generating ozone.

During the initial fast and multiple reactions of ozone with the plasmatic components, a variable amount of the ozone dose is neutralized by the wealth of the hydrophilic antioxidants. It is noteworthy that, with the exception of uric acid oxidized to allantoin, dehydroascorbate and GSH disulfide are reduced back to their normal value in less than twenty minutes due to the exceptional efficiency of the recycling system based on a multitude of reducing molecules such as alpha-lipoate, Vitamin E, thioredoxin, and last but not least NADPH [25,26] acting in a well-coordinated sequence of electron donations [27].

Most importantly, H_2_O_2_, being unionized, rapidly enters into all blood cells and the chemical gradient between plasma-cells has been measured to be about 10% of the extracellular concentration [28-30]. In other words, when the highest ozone concentration is mixed with blood, depending upon the interindividual variability of antioxidant potency (1.28-1.83 mmol/L plasma) [31], the highest H_2_O_2 _concentration measured in plasma is about 40 μM [32] and therefore inside the cells is at most 4 μM. This sudden inflow of this small amount of H_2_O_2 _inside blood cells is the indispensable stimulus to activate a series of biochemical reactions as follows:

i) in the erythrocytes: activation of glycolysis with increase of ATP and 2,3-diphosphoglycerate. Functionally, the oxyhemoglobin sigmoid curve shifts to the right and increases the release of oxygen at the tissue level. The erythrocytes mop up most of the H_2_O_2 _and promptly reduce it to water by GSH. The sudden formation of GSSG (oxidized glutathione) alters the GSH/GSSG ratio but the cell quickly correct it by either extruding some glutathione-disulphide or by reducing it via GSH reductase at the expense of either ascorbic acid or thioredoxin, which has two-SH groups. Moreover, the activation of glucose-6-phosphate dehydrogenase (G6PDH) provides reducing power and activate glycolysis.

ii) in the leukocytes: neutrophil phagocytic activity is enhanced. Inside monocytes and lymphocytes, H_2_O_2 _activates a tyrosin-kinase with consequent phosphorylation of IkB, one of the trimeric components at rest of the NF-kB [33,34]. The phosphorylated IkB detaches from the trimer and it is broken down in the proteasome. The remaining eterodimer p50-p65 is transferred into the nucleus where it can activate about 100 genes. Of great significance it is the final release of some cytokines (IFNγ and IL-8) and of some acute-phase proteins [11];

iii) in the platelets. In relation to the ozone concentration, we have measured release of PDGF-AB, TGFβ-1 and IL-8 [35]. Growth factors have a specific relevance in enhancing ulcer's healing. in peripheral arterial disease (PAD).

It must be said that the H_2_O_2 _concentration in the cells (4 μM) is essential for switching on cellular responses and it probably lasts few seconds as GSH-Pxs, peroxiredoxin and catalase promptly reduce it to H_2_O. In plasma, the H_2_O_2 _half-life is less than 1 minute and it is absent during blood reinfusion.

On the other hand, among a variety of LOPs, newly formed lipoperoxide radicals are rapidly reduced to hydroperoxide while among a variety of alkenals, the bulk originated by n-6 polyunsaturated fatty acids (PUFA) is represented by the fairly stable 4-HNE (4-Hydroxy-2-nonenal) [36], while 4-HHE is a product of n-3 PUFA [37,38]. Both aldehydes are amphipathic molecules reacting with free GSH, carnosine and mainly albumin. They eventually act as useful messengers and their toxicity is quenched by three processes schematically indicated as: a) detoxification, b) dilution, and c) excretion. Specifically:

a) Small aliquots are broken down at once by enzymes such as GSH-S-transferases and aldehyde dehydrogenase or by other detoxifying enzymes described by Awasthi et al. [39];

b) the bulk is bound to the -SH group of Cys34 present in domain-I of albumin but also to free GSH. Eleven nucleophilic residues (Lys199 and His146) can also bind up as many as eleven aldehydic molecules [40,41]. Moreover, GSH can be oxidized to a sulfonic acid while the -SH group of albumin may be oxidized to sulfenic acid [42,43].

Thus, owing to the high albumin amount (about 280 g in man), the bound alkenals undergo a great dilution in the body fluids implying a most important loss of toxicity. Alkenals have not a membrane receptor but the albumin adduct can transport it everywhere in the body.

c) Aldehydes are also excreted into bile and urine after hepatic detoxification [44] and renal excretion as mercapturic acid conjugates [45].

An interesting aspect is that albumin can transport alkenal adducts in all body tissues, from liver to endocrine glands and the central nervous system. Thus 4-HNE-Cys adducts can be released at many sites and inform a variety of cells of a transient, acute oxidative stress. At submicromolar or picomolar levels, 4-HNE can act as a well-known signaling molecule [36] able to activate the synthesis of γ-glutamate cysteine ligase, γ-glutamyl transferase, γ-glutamyl transpeptidase, HSP-70, heme-oxygenase-I (HO-1), and antioxidant enzymes such as superoxide dismutase (SOD), GSH-peroxidase, catalase and last but not least important, G6PDH, a critical enzyme electron-donor during erythropoiesis in the bone marrow. There is a wide consensus on the relevance of the induction of protective molecules during repeated oxidative stress [46-52] and it is of interest that these small stresses are of crucial importance for preventing and treating hypertension, stroke and heart infarction. Indeed in our clinical trial in peripheral arterial disease (PAD) ozonetherapy has proved to be better than the orthodox infusion of iloprost [53].

Ozone therapy is based upon a real hormetic concept where the optimal ozone dose must never overwhelm the potent antioxidant capacity of blood [54,55].

At the time of ozonated blood infusion, 4-HNE-Cys adduct can also act on the vast expanse of endothelial cells and, by stimulating the endothelial nitric oxide synthase (eNOS) enhances the biosynthesis of NO via the 5-electron oxidation of L-arginine. NO, S-nitrosothiols and a trace of CO released with bilirubin via the upregulation of HO-1 activity allows vasodilation, thus improving tissue oxygenation in ischemic tissues. Moreover, an increased production of NO counteracts the excessive endothelial release of O_2_^- ^caused by the chronic inflammation typical of atherosclerosis. Thus, both NO and CO released in trace amounts, accomplish the task of important physiological mediators. Finally, the majority of patients, undergoing several treatments, report a feeling of euphoria and a sense of wellness probably due to an improved hormonal secretion and/or better utilization of neurotransmitters. Most importantly, by using the above reported therapeutic range of ozone concentrations strictly related to the blood volume, it must be noted that neither acute nor chronic toxicity has been ever observed during or after ozone therapy [19].

In conclusion it can be stated that the M-O_3_-AHT owing to the precise volume of blood, the precise volume of ozone of which the exact concentration is photometrically determined, hence the real dose, makes it the unsurpassed method because ozone instantly reacts with several blood substrates in a quantitative and predictable fashion.

### B) Modalities of ozone administration

It is clear that ozone can be administered with great flexibility but it should never be directly injected as a gas mixture in the circulatory vessels because of the risk of provoking oxygen embolism, given the fact that the gas mixture never contains less than 95% oxygen. Schematically, the methods of ozone administrations can be classified as follows. The ozonated autohemotherapy (O_3_-AHT), distinguished in: B1) Major (M-O_3_-AHT), and B2) minor (m-O_3_-AHT), in relation to the blood volume; as well as B3) Extravascular Blood Oxygenation-Ozonation (EBOO). B4) The quasi-total body exposure (QTBE) to O_2_-O_3_. B5) The various forms of ozone administration into different tissues: i) subcutaneous (SC); ii) intramuscular (IM); iii) intradiscal (ID); iv) intracavitary (peritoneal and pleural spaces); v) intravaginal, intrauretral and vesical; vi) for dental applications, mainly as ozonated water. In detail:

B1) In the M-O_3_-AHT, a predetermined volume of blood (from 100 up to 225 mL, on the basis of the patient's body weight) to which has been added either sodium citrate 3.8% (1+ 9 mL blood) or heparin (20 IU/mL of blood) can be exposed to an equal volume of gas mixture (95%O_2_-5%O_3_), with the ozone concentration (from 20 up to 80 μg/mL of gas per mL of blood, i.e.: 0,42-1,68 mM) precisely determined by using an ozone-resistant, disposable 500 ml glass bottle under vacuum. This simple, inexpensive (all the necessary disposable material costs about 15 US$) procedure has already yielded therapeutic results in chronic limb ischemia superior to those achieved by conventional medicine [53]. It is also useful as a supporting help in chronic infection diseases and probably in autoimmune disorders.

B2) The m-O_3_-AHT is also precise and it consists in treating usually 5 mL of blood with an equal volume of gas mixture (O_2_-O_3_) with an ozone concentration of 80-100 mg/L of gas per mL of blood (total ozone dose = 0.4-0.5 mg). In this case the blood is vigorously mixed with the gas mixture for about 1 min and immediately injected in the gluteus muscle with the gas foam. It has different aims because it acts as an immune enhancer and inducer of heme-oxygenase 1 (HO-1). It is easy to perform, inexpensive, safe and well-tolerated. It supports the M-O_3_-AHT in chronic infection diseases especially useful in herpetic infections [56].

B3) The EBOO is a procedure to be used in emergency conditions such as PAD stage 4, because of complexity and invasiveness due to blood collection and infusion from two contralateral veins and blood circulation through an ozone-resistant gas exchanger [57,58]. Finally, by using a peristaltic pump, blood returns to the circulation via a contra lateral vein. About 5L of blood can be oxygenated-ozonated within one hour during which the blood/ozone quantities can be varied but always precisely determined. It has a precise rationale and normally procures a rapid improvement. However, it is expensive and must be performed by technicians specialized in extravascular blood circulation [57].

B4) The QTBE to O_2_-O_3 _for avoiding the inhalation of ozone exclude the head and the neck of the patient. On the other hand, the extensive cutaneous exposure to O_2_-O_3 _does not need any venous puncture and, owing to the vast expanse of the skin (about 1.5 m^2^) allows a generalized and beneficial effect. The usual exposure time in a perfectly insulated ozone-resistant cabinet is 20-30 min: the final ozone concentration during treatment is below 1 μg/mL delivered at both controlled and modifiable temperature (37-40°C) and Relative Humidity (≤100%) depending upon the pathology and the state of the patient. Ozone, while is never absorbed as such through the skin because it always reacts with the aqueous-lipidic cutaneous surface, allows the well-demonstrated absorption of LOPs [59]. There is no doubt that it has a pharmacological effect that, although less predictable than M-O_3_-AHT, exerts beneficial effects in cardiovascular, infectious diseases and in aging [60].

### B5) Administration of ozone into different tissues

i) In the past, O_2_-O_3 _mixture was injected into subcutaneous tissue. The ozone concentration should never exceed 20 μg/mL and the gas volume 20 mL because it elicits a transient pain and the risk of embolism must be avoided. Total multiple injections (up to 50) of 1 mL each with an ozone concentration of 2-3 μg/mL are performed as a therapy for lipodistrophy [61].

ii) Monolateral or even bilateral injection of 5-10 mL of gas with an ozone concentration of up to 20 μg/mL is performed into the trigger points of the paravertebral muscles corresponding to the metamers of the herniated disc usually interesting from L4 to S1 [62]. This "chemical acupuncture" is the indirect approach for treating lumbar disc herniation and alternate daily treatments for about 3 weeks yield a therapeutic results in about 68% of the patients [63]. Otherwise,

iii) The injection of gas (2-5 mL) with an ozone concentration of 30 μg/mL is directly performed into the herniated nucleus pulposus under radioscopic control. The percentage of cure is almost 80% [64-66];

iv) In the case of mesothelioma, peritoneal carcinomatosis or peritonitis, endoperitoneal or endopleural injection of up to 2500 mL of gaseous mixture with an ozone concentration of 10-20 μg/mL can be performed. This modality is rarely used in Western Countries and must be performed by a specialist [Bocci V, Zanardi I, Travagli V: **A rational innovative treatment for peritoneal carcinomatosis**. *Cancer Invest*. 2011, submitted];

v) Insufflation of variable volumes of gas (50-200 mL) with an ozone concentration ranging between 10-15 μg/mL of gas can be done into the vaginal, uretral and vesical cavity for different types of infections or in the case of post-radiation microhemorrhage of the bladder [67]. Vaginal washing with ozonated water (20 μg/mL) and ozonated oil pessaries are very efficacious;

vi) Primary root carious lesions are successfully treated in children by using a dental handpiece with a removable silicon cup for exposing the tooth's lesion to the gas without any leakage [68].

To summarize, ozone is characterized by versatility and the various modalities for its administration have been described. As for the clinical application, ozonated autohemotherapy is specifically suited to treat vascular diseases (e.g. stroke, peripheral arterial as well as chronic heart disease) associated with some age-degeneration (macular degeneration, dry form). It can be as a useful supportive therapy in chronic infectious diseases, diabetes and cancer, but it is not curative. On the other hand, cutaneous and ulcerative infections are excellently treated with topical application of ozonated oils and ozonated water. Such derivatives have been also used in dentistry (primary carious lesions). Finally, gaseous oxygen-ozone mixture is remarkably important in orthopedy (herniated disk).

All of these modalities, if performed by skilled hands, are very effective and practically risk-free.

Are other modalities of ozone administration safe and effective?

It is indispensable to discuss this problem because of the spreading of methodologies by inexpert and improvised ozone therapists, possibly non-physicians, can deeply damage patients and compromise the future of ozone therapy. It has become known that during the early 90's, technicians migrated into Africa and Antilles, performed direct intravenous injections of gas for treating HIV infections in desperate patients. In spite of a slow infusion, several cases of embolism and deaths have occurred. It is deplorable that even today in Western countries, certain physicians are still using this technique officially forbidden in Europe since 1984. They believe that ozone directly infused into the venous system is more effective because they do not know that ozone immediately dissolves into the plasmatic water and, owing to necessarily minute quantities, is mostly neutralized by the wealth of antioxidants. Furthermore, both intracellular and free viruses are well protected by the antioxidant system. On the other hand, the bulk of oxygen, far less soluble than ozone, forms bubbles leading to lung embolism.

However, the actual problem must be focused on the infusion of ozonated saline [69]. Although nobody can criticize the M-O_3_-AHT where equal volumes of gas and blood are precisely regulated, it must be admitted that the blood withdrawal phase, by using a G19 needle, takes about 10 minutes. As a consequence, the further 5 min mixing and about 20 min infusion into the donor imply a time period not available in hospitals where thousands of patients have to be treated every day. Indeed, since 1994 the need of a valid blood substitute was felt necessary and after an extensive search, we found that the simple physiological saline (NaCl 0.9%) could represent a possible pharmaceutical vehicle. After testing a variety of ozone concentrations, it was found that bubbling in saline gaseous mixture with an ozone concentration of 70 μg/mL for 10 min in saline could produce a level of H_2_O_2 _therapeutically acceptable. However, after the infusion of this solution in ourselves, it was experienced a painful feeling along the venous route in the infused arm during the next 24 h likely due to endothelial inflammation. A further checking revealed the transient presence of HClO as the most likely offensive agent, although Razumovskii *et al.*, by using far lower O_3 _concentration have not detected hypochlorite [70,71].

It was immediately obvious that the idea of ozonated saline had to be abandoned. However, at about the same time Russian clinicians decided that by ozonating saline with an ozone concentration as low as 2-3 μg/mL could be a viable and very quick solution to prepare and infuse every day a multitude of patients. Although it had been claimed that infusion of ozonated saline had a similar beneficial effect of M-O_3_-AHT, to the best of our knowledge no comparative clinical studies has appeared in the international literature during the last 15 years. At a recent Congress of Ozone Therapy held in Instanbul, Dr. E.I. Nazarov, President of the Ukraine Association of Ozone therapy confirmed that in Russia and Ukraine the Ministry of Health had recommended that the concentration of ozone in saline solution should not exceed 3 mg/L [69]. Consequently, Dr. Nazarov has developed a device (Bozon-N) able to automatically measure the ozone concentration in saline, establishing and supporting the same ozone concentration during the infusion. This suggests that if ozone is always present, hypochlorous acid even if in trace amount, will be also continuously generated. Although it is an excellent bactericidal compound - also produced by phagocytic cell mieloperoxidase - it exerts a deleterious reactivity with protein -SH groups, amino groups (chloramines formation) with DNA, RNA, and lipids. Moreover, ClO^- ^is one of the most noxious ROS during the chronic inflammation accompanying several pathologies.

As the infusion of ozonated saline is very cheap, minimal time-consuming than M-O_3_-AHT and quite remunerative for the practitioners, physicians have started to use it also in several Western countries and it is foreseeable that it will be extensively used in poor countries, possibly at higher ozone concentrations. Indeed, Ikonomidis *et al. *have reported that they maintain the saline solution under a constant flow of O_3 _during IV infusion but they warned that the maximum amount of O_3 _daily administered is usually 4-5 mg and should never exceed 8-10 mg [72]. In their publication they also stated "if we exceed these rates, the over coagulation syndrome starts" and they strongly recommend to perform coagulation tests before starting therapy. These warnings reinforce our preliminary objection to this approach. Moreover Foksinski *et al. *have detected 8-oxodeoxyguanosine, a typical oxidative DNA damage in lymphocytes of atherosclerotic patients after the IV infusion of ozonated saline, that is a worrisome result never detected after M-O_3_-AHT [73].

Fortunately, to the best of our knowledge, Russian physicians ozonize the saline with very low O_3 _concentrations (2-3 μg/mL) and this precaution certainly minimizes toxicity but it leaves open the aspect of therapeutic efficacy. At the infusion time, 250 mL of saline may contain about 0.5 mg of ozone, which will be entirely neutralized by antioxidants. On average M-O_3_-AHT receives about 10 mg of ozone and it is pharmacologically effective. Moreover, ozonation of saline is clearly an unstable process and therefore dangerous preparation because a pharmaco-therapeutic principle has long established on the basis of the exact knowledge of the constituents and their stability.

It is therefore necessary to enumerate and discuss the problems occurring during the preparation of ozonated saline:

1) For human use it would be unwise to use O_3 _concentration over 3 μg/mL (3 mg/L). Moreover it is essential to establish the volume per minute of the gas mixture O_2_-O_3_. The big problem is that different ozone generators present in the market have variable gas output: if it is 1 L per minute, the O_3 _delivered to 200 mL of saline would be 3 mg/L but, if the output per minute is equivalent to 3 litres of gas, then the actual dose of O_3 _delivered will be 9 mg/L! Not to mention the risk of using more than 3 μg/mL of ozone. As a consequence one must properly warn the ozone therapist in relation to the owned ozone generator as otherwise one risks to poison the patient.

2) The period of ozonation time also ought to be well defined in relation to the volume of saline because in the case of saline solution an ozonation time of 20 min appears enough to reach a plateau. Obviously a shorter or longer ozonation period will differently modify the concentration of hydrogen peroxide, O_3 _and other radicals.

3) Another aspect to be clearly defined if gas bubbling will continue or not during the IV infusion period. This is because, as soon as the gas bubbling is stopped, the concentration of H_2_O_2 _remains fairly stable but the O_3 _concentration will halve during the next 30 min and this affects the therapeutic result. As a trivial example, it is doubtful that in a large clinic all the saline infusions are all under a continuous O_3 _bubbling and it is likely that saline bottles will be ozonated and then distributed implying a more or less long delay before the infusion. After one hour delay, O_3 _is not longer present.

As a preliminary conclusion, one must seriously ponder on the validity of using the infusion of ozonated saline. It is certainly less dangerous than the direct IV infusion of the gas mixture that some doctors still dare to perform. However, it does not represent an improvement because the variable presence of H_2_O_2_, O_3_, and similar, does not insure neither a good reproducibility, nor a consistent therapeutic effect. Moreover the blood flow in the cubital vein varies considerably in different patients and in women. Consequently, a fairly constant infusion of ozonated saline versus a variable blood content of antioxidants implies an uncertain blood/H_2_O_2_-O_3 _relationship with possibly a too low or too high bio-oxidation. By comparison, a fundamental pillar of the classical M-O_3_-AHT is that we can maintain precisely the blood/O3 ratio within the known therapeutic range. In summary, the ozonated saline approach contains too many uncertain parameters and, in any case, it needs to be carefully standardized to avoid the risk of a placebo infusion or an excessive and risky treatment. Nonetheless if, on the basis of the critical need to treat too many patients, it is allowed by Russian Health Authorities, it will be never accepted by neither the FDA, USA or by the EU Health Authorities. Thus, in spite of recent studies which exclude the toxic danger of ClO^- ^formation by using no more than 3 mg/L of ozone [70,71], the use of ozonated saline remains a constant danger.

Another irrational procedure called Celacade (promoted by Vasogen Inc., Canada) consisted in exposing 10 mL of human blood to an oxygen/ozone gas mixture (ozone concentration 15.35 g/m^3^) delivered into the blood at a flow of 240 mL/min and UV light (253.7 nm) at a temperature of 42.5°C for about 20 min. The treated blood sample was removed from the system and immediately administered by intragluteal injection to the donor patient. Two treatments were given on consecutive days, followed by a third on day 14. Subsequent treatments were given at 4 week (28 days) intervals for at least 22 weeks, for a total of 8 injections.

The procedure used an expensive device able to deliver an enormously toxic dose of ozone (107.5 mg per mL of blood) plus an undetermined UV irradiation at 42.5°C. The final ozone dose is about 15000-fold higher than the average ozone dose used during the classical O_3_-AHT and the extremely high oxidation of blood causes a complete denaturation of blood components. This procedure was invented aiming to establish a non-specific immunomodulation therapy (IMT) in the hope of reducing the inflammatory process and the chronic oxidative stress present in vascular diseases. It has proved to be useless in a multicenter, randomized, double-blind, placebo-controlled study in 533 patients with symptomatic peripheral arterial disease (PAD), called the SIMPADICO trial [74]. It is most important noting that this trial had to be stopped three months early because it did not show any improvement in PAD and caused a significantly higher rate of malignancies in the IMT group [75]. Moreover, a subsequent multicenter study in 2426 patients, called the ACCLAIM trial, in chronic heart failure resulted in a "disappointing" results [76]. Thus almost 3000 patients with critical vascular pathologies have undergone a useless and potentially dangerous procedure. Moreover, in spite of two commentaries reprimanding the EMA [77,78], it seems that the ozone-UV-heat apparatus remains on sale in Europe.

Another route of administration of ozone, firstly advocated by Dr. Aubourg is the insufflations of O_2_-O_3 _gaseous mixture into the rectum for treating chronic colitis and fistulae [79]. In 1936 this was a reasonable application which it has been now extended to treat all diseases. The insufflations of a volume of 200-300 mL of gas into the rectum-colon at ozone concentration ranging from 5 to no more than 35 μg/mL can be precisely done but it remains unpredictable as an effective dose of ozone because of a possible flatulence and the presence of a more or less abundant luminal content. Thus, it is obvious to predict that a significant fraction of the dose will be neutralized by fecal material. The residual dose of ozone will dissolve and be neutralized into the layer composed of glycocalix and mucoproteins covering the mucosa. Ozone will instantly and fully react with these compounds but only a LOPs fraction will be absorbed with O_2 _by the mucosa. Indeed, in a rabbit experiment we have shown a transient presence of LOPs in the portal vein [80], but obviously the real pharmacological effect on vascular diseases and diabetes remains uncertain.

Once again, while in Western countries many patients object to this route, at Cuba they have thousands of patients to be treated every day and they have adopted this rapid, inexpensive procedure in all patients always administering 200 mL of gaseous O_2_-O_3 _mixture with the excessive ozone concentration of 50 μg/mL (ozone dose: 10 mg). Firstly, the ozone concentration is too high and during prolonged use may be mutagenic; secondly, this route, being so uncertain, should not be used in controlled clinical trials. Indeed, it seems that in only twenty days that dosage could cure (?) the diabetic foot in a number of patients treated with rectal ozone and topical ozonated oil [81].

Finally, owing to the fact that H_2_O_2 _is one of the most important ROS generated by O_3_, since 2005 [82], in women with very difficult venous access, by using a G27 needle, we have intravenously infused the solution of pure H_2_O_2 _in glucose (5%) or saline solutions at the concentrations ranging from 0.03-0.06% (8-16 mM). The bio-oxidative therapy with H_2_O_2 _was first described by Dr. I.N. Love in 1888 [83] and then promoted by Dr. C.H. Farr in 1993 [84]. We showed a modest but consistent activity in women with age-related macular degeneration. In contrast to ozonated saline, this compromise, very simple to prepare, does not contain other dangerous ROS and moreover there is no need for ozone generators. This solution has the further advantage to be possibly used in remote African and Asian areas. Obviously, the glucose solution should not be used in diabetic patients but H_2_O_2 _can be dissolved in physiological solution and used at once.

## Conclusions and perspectives

The application of ozone in medicine represents one of the most intriguing adventures in research. First of all because ozone is too well known as a toxic gas in the troposphere and secondly, in spite of courageous pioneers like Payr, Fisch, Wolff and Auborg, ozone stalled in an empirical phase for almost three decades and it was heavily damaged by the improper use of direct IV infusion by so-called doctors in desperate HIV-AIDS patients. This dangerous and useless application, associated with other deplorable episodes, led the FDA to prohibit the use of ozone in medicine. Today, the basic mechanisms of action of ozone in blood, documenting the existence of a therapeutic window and establishing a framework for understanding and recommending ozone therapy in some diseases have been clarified and we can start to see a faint light at the end of the tunnel. However, owing to the lack of sponsors and funding, the clinical work, fundamental for demonstrating the validity of ozone therapy, proceeds at a snail pace. Furthermore, the urgent need of treating too many patients in poor countries has stimulated the use of "cheap and quick" procedures that hinder progress. Clinical scientists for either prejudice, or lack of knowledge, or exclusive interest in pharmaceutical drugs disregard, if not object, ozone therapy that remains in the hands of practitioners who cannot deliver reliable scientific reports. Thus, optimistically let us say that we are at the end of the beginning but our enthusiasm remains high and we will continue our efforts for allowing the acceptance of ozone therapy as an efficacious approach to be included among the armamentarium of orthodox medicine.

## Competing interests

The authors declare that they have no competing interests.

## Authors' contributions

VB and VT conceived, outlined the direction of, provided information to shape the manuscript content and discussion, gathered references, and drafted the manuscript. IZ refined the search for information and gathered references. All authors read and approved the final manuscript.
